# Multimodal Reconstructive Treatment of the Face and Scalp Following Catastrophic Burn Injury: An Eight-Year Experience with Structural Fat Grafting, Follicular Unit Excision Hair Transplantation, and Scalp Micropigmentation

**DOI:** 10.3390/jcm15166227

**Published:** 2026-08-12

**Authors:** Jerzy Roch Kolasinski

**Affiliations:** Klinika Kolasinski, 62-020 Swarzedz, Poland; colas@klinikakolasinski.pl

**Keywords:** burn reconstruction, face, scalp, FUE hair transplantation, fat grafting, scalp micropigmentation, alopecia, scar, cicatricial alopecia

## Abstract

**Background:** Severe burn injuries involving the face and scalp have profound physical and psychosocial consequences, and represent the greatest reconstructive challenge in plastic surgery. One comprehensive solution involves the sequential application of autologous fat grafting, follicular unit excision (FUE) hair transplantation, and scalp micropigmentation (SMP); however, the long-term outcomes of this approach have rarely been documented. **Methods:** A male patient sustained catastrophic methane explosion burns at age 27, involving 79% total body surface area (TBSA) with full-thickness involvement of the face and scalp (Revised Baux Score 123; estimated mortality risk 50–80%). Following 65 reconstructive procedures at external institutions over six years, he was referred to our center at age 30. He presented with extensive facial and scalp scarring, complete absence of eyebrow, beard, and mustache hair, and widespread scalp alopecia. Over an eight-year period, the patient received 11 sessions of autologous FUE hair transplantation (7180 follicular units to the eyebrows, beard, and scalp), four sessions of structural fat grafting using the Coleman technique, and five sessions of SMP. **Results:** The patient achieved complete reconstruction of facial hair, a natural scalp hairline, and significant scar quality improvement. **Conclusions:** Our findings demonstrate that systematic, long-term multimodal reconstruction, i.e., combining fat grafting, FUE transplantation, and SMP, can yield clinically meaningful esthetic and psychosocial outcomes, even after the most catastrophic burn injuries.

## 1. Introduction

Burn injuries remain a global burden on public health, accounting for an estimated 11 million injuries and approximately 180,000 deaths annually [1]. In Europe, the incidence of severe burns requiring hospitalization is estimated at 0.2–2.9 per 10,000 population per year, with many of the worst cases being associated with industrial and occupational accidents such as coal mining and chemical spills [2]. In many centers, burns affecting more than 40% TBSA (total body surface area) are associated with mortality rates of over 50%, and injuries involving more than 70% TBSA can present a challenge for even the most experienced burn units [3].

The damage associated with major thermal injury extends far beyond local tissue destruction. Full-thickness burns destroy the epidermis, dermis, and subcutaneous tissue, resulting in the permanent loss of associated structures, such as sweat glands and sebaceous glands. In addition, in the head and neck areas, scarring can cause cicatricial ectropion, which threatens corneal integrity, as well as perioral contracture, which impairs nutrition and speech, and cervical contracture, which restricts head movement. Damage to the scalp can also result in permanent alopecia, which has a strong influence on appearance and social identity [4,5].

Recent decades have seen a considerable expansion in the reconstructive armamentarium available for post-burn sequelae of the head and neck. The range of modern techniques now include local and regional flaps, split-thickness skin grafting, autologous fat grafting, autologous hair transplantation, and scalp micropigmentation [6,7,8]. The present study describes the case of a patient with catastrophic pan-facial and scalp burn sequelae, who received sequential application of various treatments over an eight-year period. Its findings demonstrate the restorative potential of such systematic, long-term reconstructive programs.

## 2. Case Presentation

A 30-year-old male patient with extensive scarring of the head region was admitted to our institution on 18 October 2017 for reconstructive treatment of the hair-bearing areas of the face and scalp.

**Figure 1:**
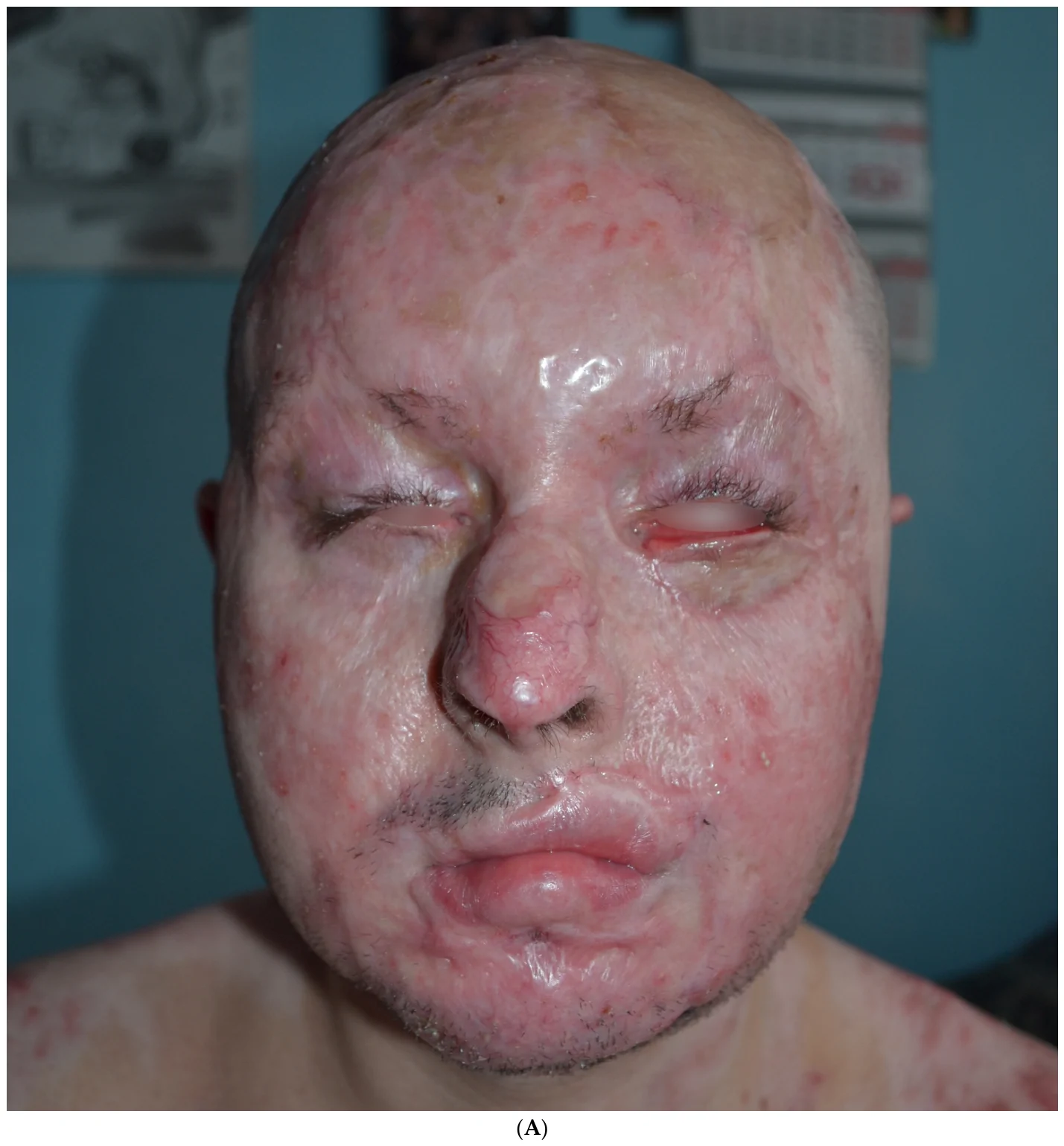

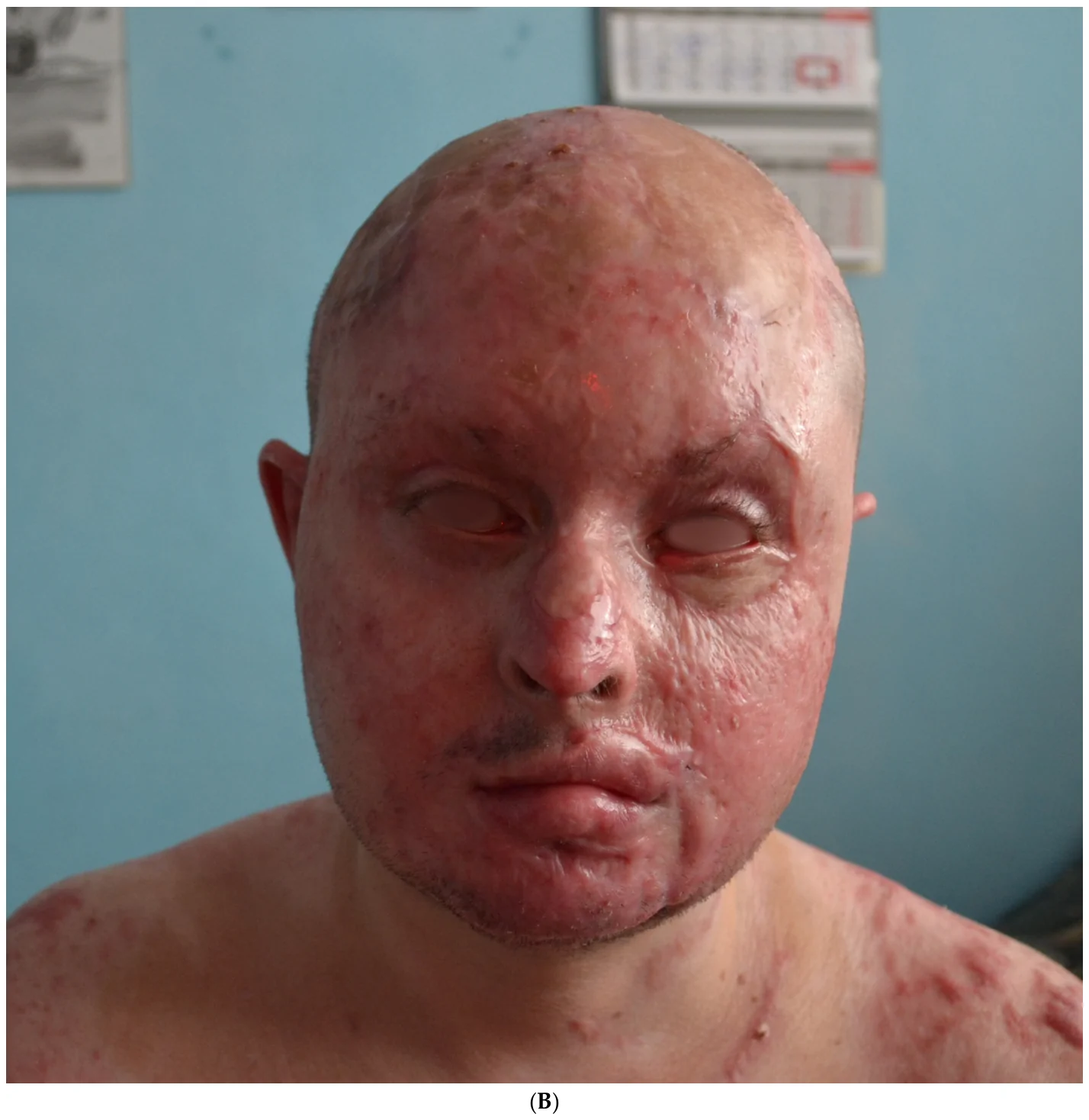
Acute post-burn state during initial hospitalization (Burn Treatment Center, Siemianowice Śląskie, October 2014). (**A**) Early post-burn phase. (**B**) Subacute phase. These images illustrate the severity of the initial injury.

The patient received the injuries as a result of thermal trauma caused by a methane explosion in a coal mine on 7 October 2014. Immediately after the accident, he was admitted to a regional burn center in critical condition, with deep dermal/full-thickness (IIb/III degree) thermal burns involving approximately 79% total body surface area (TBSA): this area included the face, head, neck, chest, trunk, buttocks, abdomen, perineum, and both upper and lower extremities (Figure 1). The patient also experienced eyelid insufficiency due to facial burns to the eyelids, conjunctivae, and corneas of both eyes. Inhalation injury was also present. Given the extent and depth of burns, the patient’s Revised Baux Score was approximately 123, corresponding to an estimated mortality risk of 50–80% [9].

Over the following six years, the patient underwent 65 reconstructive procedures at five external institutions. The complete procedural list is provided in Supplementary Materials, Table S1.

**Figure 2:**
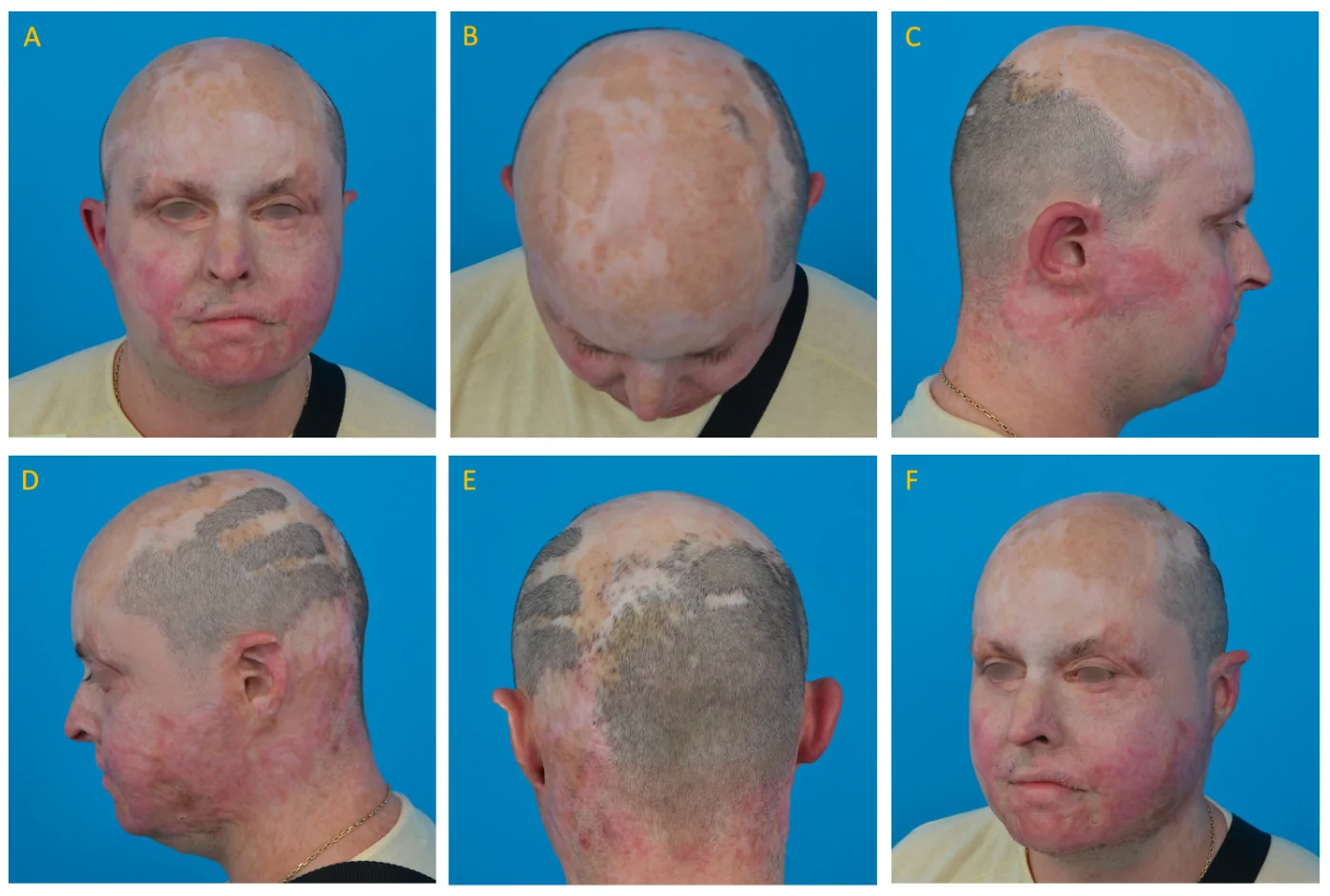
Patient at initial presentation to our institution (October 2017). (**A**) Frontal view. (**B**) Superior (vertex) view. (**C**) Right lateral view. (**D**) Left lateral view. (**E**) Posterior view. (**F**) Right oblique view.

On admission to our institution on 18 October 2017, the patient presented with widespread, rigid facial and scalp scars, and complete loss of hair from the eyebrows, mustache, and beard areas. The temporal, frontal, parietal, and partially left occipital regions of the scalp were covered with split-thickness skin grafts. The temporal and occipital areas also presented irregular areas of residual hair-bearing skin (Figure 2). The patient also reported experiencing depressive symptoms, characterized by a ruminative, past-focused cognitive style and markedly reduced engagement with present and future life circumstances.

The patient underwent 11 follicular unit excision (FUE) sessions, during which a total of 7180 follicular units (FUs) were transplanted. In the eyebrow region, two sessions were performed with a cumulative total of 490 FU; single- and double-hair follicular units were used to reconstruct the natural eyebrow architecture. For the mustache and beard, four sessions were performed with a cumulative total of 2300 FU. For the scalp, five sessions were performed with a cumulative total of 4390 FU. Donor follicles were harvested from the remaining hair-bearing occipital and temporal scalp using 0.9 mm FUE hybrid punches and the WAW system under local anesthesia. Graft implantation was performed using the stick-and-place technique.

Four sessions of structural fat grafting were performed in the areas of facial and scalp scarring. Adipose tissue was harvested by manual lipoaspiration from the abdominal wall and centrifuged (Coleman technique); the resulting material was then injected into the subdermal and intradermal planes of the scar tissue using blunt-tipped microcannulas in a retrograde fanning pattern [10,11]. Each fat grafting session was performed following a six-month interval after the hair transplant procedure. Where scalp scars were present, fat tissue was injected directly beneath the split-thickness skin graft. Fat grafting was also performed into facial scars, which were subsequently covered with hair transplants, and into the cheek and neck areas, which were not; in both cases, the scars became significantly more pliable and smoother, and any contractures were resolved. No complications were observed following either fat grafting or hair follicle transplantation.

To replicate the appearance of shaved follicular units, the FUE transplantation was complemented by five scalp micropigmentation (SMP) sessions. Pigment was deposited at the level of the papillary dermis within alopecic or low-density areas of the scalp using a specialized digital device with a fine needle.

**Figure 3:**
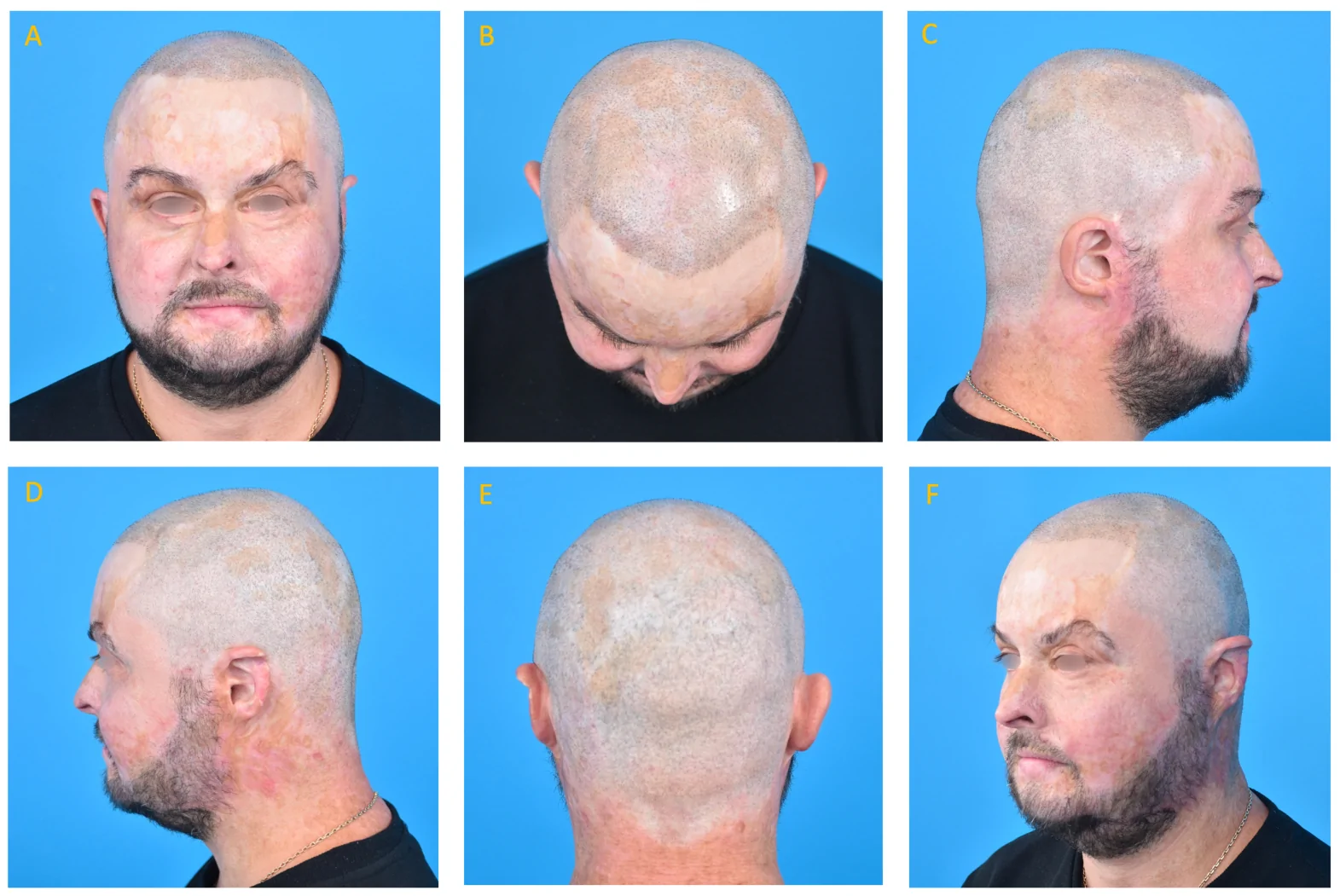
Patient following completion of the eight-year reconstructive program at our institution (September 2025). (**A**) Frontal view. (**B**) Superior (vertex) view. (**C**) Right lateral view. (**D**) Left lateral view. (**E**) Posterior view. (**F**) Right oblique view.

Treatment at our institution was concluded on 16 September 2025 with complete reconstruction of the eyebrow, mustache, and beard hair. The resulting facial hair pattern significantly improved facial identity and gender-appropriate appearance (Figure 3). The patient re-engaged fully in professional and family life: during the treatment period, he became the father of two children. He also reported general improvements in social reintegration and quality of life, although it should be noted that these observations remain subjective.

## 3. Discussion

The present report describes the case of a patient who had sustained burns involving 79% TBSA, with an estimated mortality risk of 50 to 80% on the Revised Baux Score [3,9]. His recovery demanded extraordinary clinical resources and the coordinated efforts of specialists and surgeons from burns intensive care units, together with a multidisciplinary team. As a result, the patient had undergone 65 separate procedures at external institutions over the six years preceding his arrival at our center.

Full-thickness burns of the face and scalp carry a unique selection of long-term physical sequelae. The presence of high amounts of graft-covered scar tissue over the face results in a loss of dynamic facial expressiveness, reduces cutaneous sensibility and prevents sebaceous secretion. These features, together with the characteristic inelastic appearance of the face, are characteristic of patients with severe burns [7]. Perioral scarring impairs oral opening, chewing, and speech; in addition, cervical contracture restricts head movement and poses a challenges for airway management [12]. From the esthetic and social perspectives, these effects are further exacerbated by the loss of hair from the scalp, eyebrows, or beard.

Catastrophic burn injury is known to confer a psychological burden to the patient. Post-traumatic stress disorder (PTSD) has been reported in 30–45% of major burn survivors, with the risk increasing with burn size and facial involvement [13]. Also, an estimated 30–50% of survivors report experiencing depression during the first year after injury [14]. Indeed, as commonly noted in major burn survivors in the late reconstruction phase, the patient described in the present case was characterized by a clinically evident depressive symptomatology and a ruminative, past-focused cognitive style.

However, following the procedure, the patient reported improved social reintegration and quality of life. The successful reconstruction of his eyebrows, beard, and scalp appeared to act as a turning point in self-perception and social engagement. Indeed, facial hair restoration is often perceived by burn survivors as having one of the greatest psychosocial impacts of all reconstructive interventions [15]. It should be noted that the subjective psychological improvement reported by the patient was not supported by any objective data: no tests on mental state or quality of life were administered before or after treatment.

When planning the reconstructive strategy, care was taken to optimize the esthetic and psychological benefits while respecting the biological limitations of the available tissue. Therefore, structural fat grafting was included as a preparatory step to assist FUE and transplantation into scarred recipient beds; fat grafts improve scar quality and normalize the dermal environment by reducing skin rigidity and improving vascularity [16,17]. The benefits of fat grafting have been attributed to the properties of the adipose-derived stem cells (ADSCs) contained within the graft; they have been found to reduce fibroblast activity, reduce excessive collagen deposition, and promote ECM remodeling by secreting inter alia TGF-β, matrix metalloproteinases (MMPs), and IL-10. In addition, recent research has found ADSC-derived exosomal miRNA miR-125b-5p to suppress Smad2 signaling in hypertrophic scar fibroblasts, reducing collagen I and III deposition and attenuating pathological fibrosis [18]. Fat grafting has been demonstrated to significantly improve scar pliability, vascularity, and pigmentation in burn-related scars, attributing these outcomes largely to the paracrine activity of ADSCs and their pro-angiogenic effects [19].

During FUE, the best-quality follicular units are selectively harvested from donor zones; in this case, the most socially visible deficit was addressed first, i.e., the eyebrows, followed by the beard area, then the scalp [20,21]. The scalp presented poorer quality was treated by repeated fat grafting [10,11]. In the present case, correct graft distribution was ensured by employing the stick-and-place technique, which was performed by the senior surgeon; this approach also avoided poor graft take, infection, and cyst formation.

When the transplanted hair had matured sufficiently to assess any deficits in residual density, scalp micropigmentation was introduced as a “substitute” for hair transplantation: the aim of the procedure was not to replace hair transplantation, but to improve its appearance by filling out the follicular units and defining the hairline contour [22]. In the present case, micropigmentation was necessary as the donor area was limited, and it was not possible to harvest a sufficient number of grafts to achieve a satisfactory hair density in the recipient area. Also, micropigmentation allowed for a more uniform appearance by reducing the contrast between the right temporal and occipital area, with originally dense hair growth, and the left occipital area, which exhibited lower hair density due to the burn injury.

## 4. Conclusions

This case documents the eight-year reconstructive journey of a patient who survived catastrophic burn injuries involving 79% TBSA, with extensive full-thickness involvement of the face and scalp. The patient ultimately achieved complete restoration of facial hair, a natural scalp hairline, improved scar quality, and full psychosocial recovery.

This outcome was made possible through the sequential application of complementary modalities, viz. fat grafting, FUE hair transplantation, and scalp micropigmentation, and their synergic effects. However, in such cases, the true measure of reconstructive success is the return of a severely burned patient to professional life and family engagement.

## Data Availability

No new datasets were generated or analyzed in this study. The clinical data supporting the findings are contained within the article.

## Supplementary Materials

The following supporting information can be downloaded at: https://www.mdpi.com/article/10.3390/jcm15166227/s1, Table S1: Complete List of Reconstructive Procedures Performed at External Institutions Prior to Referral to Our Institution.
