# Supplementary material for: Multimodal Reconstructive Treatment of the Face and Scalp Following Catastrophic Burn Injury: An Eight-Year Experience with Structural Fat Grafting, Follicular Unit Excision Hair Transplantation, and Scalp Micropigmentation
Source: J Clin Med. 2026 Aug 12;15(16):6227. doi: 10.3390/jcm15166227 (PMC13513320; doi:10.3390/jcm15166227)
Supplement: Supplementary file 1 [file jcm-15-06227-s001.zip › jcm-4453780-supplementary.pdf]

Supplementary Materials

Multimodal Reconstructive Treatment of the Face and Scalp Following Catastrophic Burn Injury: An Eight-Year Experience with Structural Fat Grafting, Follicular Unit Excision Hair Transplantation, and Scalp Micropigmentation

Jerzy Roch Kolasinski

Table S1. Complete List of Reconstructive Procedures Performed at External Institutions Prior to Referral to Our Institution.

No.	Date of Stay	Procedures During Hospitalization	Institution
1	2014-10-07<br>to<br>2014-11-19	2014-10-07<br>1. Escharotomy of both upper extremities.<br>2. Application of xenogenic skin to wounds after escharotomy.<br>3. Harvesting of skin for culture of autologous keratinocytes.<br><br>2014-10-08<br>4. Release of the median nerve of the right upper extremity.<br>5. Decompressive incisions of the anterior surface of the left forearm.<br>6. Decompressive incisions of the anteromedial surface of the distal third of the left arm.<br>7. Coverage of incision areas of the left upper extremity with xenogenic tissue.<br><br>2014-10-14<br>8. Tangential excision of necrosis and debridement of wounds of the forearms, thighs, knees, and chest.<br>9. Coverage of forearm wounds with 1:3 meshed skin grafts harvested from both lower legs.<br><br>2014-10-27<br>10. Demarcation and debridement of wounds of the head, face, neck, upper and lower extremities.<br>11. Coverage of facial and upper extremity wounds with full-thickness and 1:3 meshed grafts harvested from the chest and hips.<br>12. Coverage of upper and lower extremity wounds with allogeneic skin.<br><br>2014-11-05<br>13. Demarcation and debridement of wounds of the head and right auricular region.<br>14. Demarcation and debridement of wounds of the face, arms, forearms, and hands; coverage with cultured fibroblasts and keratinocytes in leukocyte- and platelet-rich concentrate, split-thickness free skin grafts from hips and thighs, and allogeneic skin grafts.	Intensive Care Unit, Burn Treatment Center, Siemianowice Śląskie

		2014-11-09<br>15. Debridement of wounds of the chest, back, upper and lower extremities.<br><br>2014-11-17<br>16. Debridement of facial wounds and application of allogeneic amniotic membrane grafts.<br><br>2014-11-19<br>17. Demarcation of necrotic tissues of the fingers of both hands.<br>18. Stabilization of the fingers with Kirschner wires.<br>19. Debridement of wounds of the hands, forearms, face, and head.<br>20. Harvesting of split-thickness free skin grafts from the thighs, lateral chest wall, right buttock, and back.<br>21. Coverage of debrided wounds with full-thickness and 1:2 meshed skin grafts.	
2	2014-11-19<br>to<br>2015-02-03	2014-12-02<br>22. Debridement of granulating wounds of the upper extremities.<br>23. Application of split-thickness free skin grafts (full-thickness and meshed 1:1.5).<br>24. Drainage of subcutaneous purulent collections of the head.<br><br>2014-12-15<br>25. Debridement of granulating wounds of the face, auricles, and head.<br>26. Coverage of debrided wounds with full-thickness skin grafts.<br><br>2014-12-23<br>27. Incision below the eyelid margin of the left eyelid, correction of eyelid eversion, tarsorrhaphy traction.<br>28. Full-thickness free skin graft from the left supraclavicular region to the eyelid defect.<br><br>2015-01-02<br>29. Debridement of post-burn granulating wounds of the cranial region with split-thickness free skin grafts.<br>30. Full-thickness free skin graft to the stump of the left fourth finger (spontaneous amputation of distal phalanx).<br>31. Removal of Kirschner wires from right fingers III and IV; amputation of distal phalanx of right finger V.<br>32. Debridement of post-burn granulating wounds of dorsal distal phalanges of fingers I–IV of the right hand.<br>33. Full-thickness free skin grafts to the tips of debrided distal phalanges of fingers I–IV of the right hand.<br>34. Debridement of the wound at the olecranon of the right ulna.	Surgery Department, Burn Treatment Center, Siemianowice Śląskie
3	2015-03-19<br>to<br>2015-03-22	35. Placement of an amniotic membrane graft on the left cornea.	Ophthalmology Department, Provincial Specialist Hospital No. 5 (St. Barbara), Sosnowiec
4	2015-04-01<br>to<br>2015-04-06	36. Release of cicatricial contracture of the left eyelid using a free skin graft.	Department of Plastic Surgery, Specialist Medical Center, Polanica Zdrój
5	2015-04-27<br>to<br>2015-05-02	37. Release of contracture of the upper and lower eyelids and medial canthus of the left eye using a free skin graft.	Department of Plastic Surgery, Specialist Medical Center, Polanica Zdrój
6	2015-07-22<br>to<br>2015-07-27	38. Separation of post-burn syndactyly between the I and III interdigital spaces of the right hand.	Department of Plastic Surgery, Specialist Medical Center, Polanica Zdrój
7	2015-09-28<br>to<br>2015-10-02	39. Separation of post-burn syndactyly of the second and fourth interdigital spaces of the right hand using a full-thickness free skin graft harvested from the inguinal region.	Department of Plastic Surgery, Specialist Medical Center, Polanica Zdrój
8	2016-01-11	40. Separation of post-burn syndactyly of the III interdigital space of	Department of Plastic

	to<br>2016-01-15	the left hand using a free skin graft.<br>41. Deepening of the I interdigital space of the left hand.	Surgery, Specialist Medical Center, Polanica Zdrój
9	2016-05-25<br>to<br>2016-05-26	42. Deepening of the first and fourth interdigital spaces of the right hand.<br>43. Release of contracture of the fifth ray and dorsal metacarpus using multiple Z-plasty.<br>44. Excision of residual nail matrices of fingers I–IV.<br>45. Corrective wedge osteotomy with arthrodesis of the proximal interphalangeal joints of fingers II–IV.	Department of Plastic Surgery, CenterMed Hospital, Kraków
10	2016-10-09<br>to<br>2016-10-11	46. Laser correction of hypertrophic scars of the face and upper extremities using CO_2 laser (general anesthesia).	Surgery Department, Burn Treatment Center, Siemianowice Śląskie
11	2016-11-30	47. Laser correction of scars on the face, upper extremities, and trunk using CO_2 laser.<br>48. Administration of platelet-rich plasma to the face, upper extremities, and trunk.	DermaMed Center for Aesthetic Medicine and Laser Therapy, Wrocław
12	2017-01-04	49. Laser correction of scars on the face, upper extremities, and trunk using CO_2 laser.<br>50. Administration of platelet-rich plasma to the face, upper extremities, and trunk.	DermaMed Center for Aesthetic Medicine and Laser Therapy, Wrocław
13	2017-01-15<br>to<br>2017-01-18	51. Release of thumb contracture and separation of syndactyly between fingers II–III and IV–V of the left hand.	Department of Plastic and Reconstructive Surgery, Małopolska Burn and Plastic Center, Specialist Hospital, Kraków
14	2017-02-22	52. Laser correction of scars on the face, upper extremities, and trunk using CO_2 laser.<br>53. Administration of platelet-rich plasma to the face, upper extremities, and trunk.	DermaMed Center for Aesthetic Medicine and Laser Therapy, Wrocław
15	2017-02-29	54. Laser correction of scars on the face, upper extremities, and trunk using CO_2 laser.<br>55. Administration of platelet-rich plasma to the face, upper extremities, and trunk.	DermaMed Center for Aesthetic Medicine and Laser Therapy, Wrocław
16	2017-06-22<br>to<br>2017-06-25	56. Release of post-burn contracture of the fifth finger.<br>57. Reconstruction of the extensor tendon of the fourth finger of the left hand.<br>58. Correction of scars on the left auricle and face.	Department of Plastic Surgery, CenterMed Hospital, Kraków
17	2017-10-12<br>to<br>2017-10-14	59. Laser correction of hypertrophic scars on the forearms and hands using CO_2 laser (general anesthesia).	Surgery Department, Burn Treatment Center, Siemianowice Śląskie
18	2018-02-21	60. Laser correction of scars on the face, upper and lower extremities, and trunk using CO_2 laser.	DermaMed Center for Aesthetic Medicine and Laser Therapy, Wrocław
19	2018-04-04	61. Laser correction of scars on the face, upper extremities, chest, and abdomen using CO_2 laser.<br>62. Administration of platelet-rich plasma to the facial area.	DermaMed Center for Aesthetic Medicine and Laser Therapy, Wrocław
20	2019-01-14<br>to<br>2019-01-17	63. Laser correction of scars on the upper extremities (general anesthesia).	Surgery Department, Burn Treatment Center, Siemianowice Śląskie
21	2020-12-19<br>to<br>2020-12-31	64. Laser correction of scars on the upper left extremity (general anesthesia).	Surgery Department, Burn Treatment Center, Siemianowice Śląskie
22	2021-01-20<br>to<br>2021-01-22	65. Laser correction of scars on the right hand and forearm (general anesthesia).	Surgery Department, Burn Treatment Center, Siemianowice Śląskie

STSG, split-thickness skin graft; FTSG, full-thickness skin graft; FUE, follicular unit excision.
